# Identification of Multipath Genes Differentially Expressed in Pathway-Targeted Microarrays in Zebrafish Infected and Surviving Spring Viremia Carp Virus (SVCV) Suggest Preventive Drug Candidates

**DOI:** 10.1371/journal.pone.0073553

**Published:** 2013-09-12

**Authors:** Paloma Encinas, Pablo Garcia-Valtanen, Blanca Chinchilla, Eduardo Gomez-Casado, Amparo Estepa, Julio Coll

**Affiliations:** 1 Department of Biotechnology, Instituto Nacional Investigaciones Agrarias (INIA), Madrid, Spain; 2 Department of Biochemistry, Universidad Miguel Hernández, Elche (UMH), Alicante, Spain; University of North Carolina at Chapel Hill, United States of America

## Abstract

Spring viremia carp virus (SVCV) is a rhabdovirus seasonally affecting warm-water cyprinid fish farming causing high impacts in worldwide economy. Because of the lack of effective preventive treatments, the identification of multipath genes involved in SVCV infection might be an alternative to explore the possibilities of using drugs for seasonal prevention of this fish disease. Because the zebrafish (*Danio rerio*) is a cyprinid susceptible to SVCV and their genetics and genome sequence are well advanced, it has been chosen as a model for SVCV infections. We have used newly designed pathway-targeted microarrays 3-4-fold enriched for immune/infection functional-relevant probes by using zebrafish orthologous to human genes from selected pathways of the Kyoto Encyclopedia of Genes and Genomes (KEGG). The comparative analysis of differential expression of genes through 20 pathways in 2-day exposed or 30-day survivors of SVCV infection allowed the identification of 16 multipath genes common to more than 6 pathways. In addition, receptors (Toll-like, B-cell, T-cell, RIG1-like) as well as viral RNA infection pathways were identified as the most important human-like pathways targeted by SVCV infection. Furthermore, by using bioinformatic tools to compare the promoter sequences corresponding to up and downregulated multipath gene groups, we identified putative common transcription factors which might be controlling such responses in a coordinated manner. Possible drug candidates to be tested in fish, can be identified now through search of data bases among those associated with the human orthologous to the zebrafish multipath genes. With the use of pathway-targeted microarrays, we identified some of the most important genes and transcription factors which might be implicated in viral shutoff and/or host survival responses after SVCV infection. These results could contribute to develop novel drug-based prevention methods and consolidate the zebrafish/SVCV as a model for vertebrate viral diseases.

## Introduction

Viral infections including those caused by rhabdoviruses remain one of the most devastating diseases in fish farming. The spring viremia carp virus (SVCV), for example, causes a seasonal disease strongly affecting warm-water cyprinid fish farming in central Europe [1,2].

Although efficient prevention methods for fish diseases, such as DNA vaccines, could be used theoretically for any fish pathogen, in practice, many DNA vaccines do not perform satisfactorily for related viruses. For instance, DNA vaccines work well for *novirhabdoviruses* (those fish *rhabdoviruses* which code for a non-viral NV protein) but not for SVCV which is a non-*novirhabdovirus* [3,4]. Therefore, one exciting field of research is to study the difference between these two rhabdoviral models of fish infection/vaccination. On the other hand, more basic knowledge on fish immune responses is required to move existing DNA vaccines to *novirhabdoviruses* from injectable to more practical oral immunizations [5,6]. Studies using microarrays could greatly contribute to fill out this basic knowledge and might also contribute to improve other fish DNA vaccines [7]. Because neither the most important immediate gene targets nor the genes implicated in survival to viral infections [8,9] are well known, the potential use of drugs for seasonal prevention of fish viral infections have not been developed.

The zebrafish *Danio rerio* is a suitable model to study SVCV infections because zebrafish is a cyprinid susceptible to SVCV, their genome sequence and genetics are well known and there is no viral disease naturally affecting zebrafish [10,11]. In addition, because zebrafish are susceptible not only to *rhabdoviruses* lacking a NV gene and affecting warm-water fish such as SVCV [11,12] but also to *novirhabdoviruses* coding for NV and affecting cold-water fish such as viral haemorrhagic septicemia (VHSV) [13], infectious hematopoietic necrosis (IHNV) [14] or snake-head rhabdovirus (SHRV) [15], zebrafish can be used for comparative studies between NV-lacking and NV-coding fish rhabdoviral groups. VHSV was chosen in a previous zebrafish microarray study [16] because both infection-by-immersion (the natural route of infection) in both larvae [12] or adults [13] and successful vaccination had been described for this *novirhabdovirus* [13]. However, zebrafish acclimation to lower than their optimal temperatures (~14 °C) was required for successful VHSV infection, therefore making SVCV infections (~ 24°C) effects easier to interpret without any interference of possible low temperatures.

The use of microarrays to study fish transcriptomic changes after immunization with fish rhabdoviruses lacking the NV gene, such as SVCV [17,18], have not been described yet [9,12]. In contrast, the expression profiling of thousands of fish genes have been estimated with wide-genome microarrays during infections with *Novirhabdoviruses* such as VHSV [16,19-21], IHNV [22,23] and Hirame rhabdovirus (HRV) [24] as previously reviewed [25]. More recently, mass sequencing methods such as those using 454-pyrosequencing, have been applied also to the VHSV/turbot model [26]. In most of the above mentioned studies [19,20,22-24,27-30], the maximal number of significantly expressed fish genes was detected 2-3 days after infection. Accordingly to all these reports, we have focused our present work in the zebrafish responses to immediate infection (2-days) and compare them to those from survivor fish (30-days). On the other hand, a targeted- rather than a wide-genome microarray strategy (used in all previous reports) was chosen for this work because of the abundant annotated zebrafish genes. By targeting smaller groups of functionality focused data we also expected to enhance the link of the results to the most important immune-related genes to suggest potential drugs for prevention. Furthermore, because of their smaller size, targeted microarrays admit more replicates per array and more microarrays per slide, thus increasing reproducibility/significance of the data and lowering the costs per sample, respectively. Although not discovering any new sequences, the targeted strategy also favors interpretation of the results and requires no further annotation efforts and simpler bioinformatic data calculations.

Therefore, we chose to investigate which host genes were the most implicated in the SVCV/zebrafish infection model to explore the possible existence of associated drugs to hypothetically prevent this disease. Because the immune response depends of a complex interrelation of different proteins acting synergistically across different pathways through common genes to promote survival, we study those potentially relevant pathways. Assuming that human gene pathways were similar to those of zebrafish, we used the Kyoto Encyclopedia of Genes and Genomes (KEGG) to design a zebrafish pathway-targeted microarray by using annotated orthologous from human immune/infection pathways. The comparative analysis of SVCV-dependent zebrafish gene differential expression through 20 pathways in 2-day exposed or 30-day survivors of infection identified 16 genes common to > 6 pathways (multipath genes). In addition, receptor (Toll-like, B-cell, T-cell, RIG1-like) as well as viral RNA infection pathways were identified as the most important targeted pathways for SVCV infection. Furthermore, by using bioinformatic tools to compare the promoters of up and downregulated multipath genes, we identified putative common transcription factors which might be controlling such multiple gene responses in a coordinated manner. Known drug candidates described for multipath gene human orthologous could be identified through searching of existing databases and might be proposed for future research on its potential to develop new drugs for prevention of SVCV seasonal outbreaks. Thus the use of pathway-targeted microarrays allowed for the identification of hypothetical most important target genes and transcription factors implicated in host survival and/or viral shutoff responses after SVCV infection. These results could contribute to develop novel prevention drug-based methods and consolidate the zebrafish/SVCV model to study other vertebrate viral diseases.

## Materials and Methods

### ZF4 cell culture and SVCV virus

Zebrafish embryonic fibroblast ZF4 cells [31] purchased from the American Type Culture Collection (ATCC number CRL-2050) were used in this work. ZF4 cell lines were maintained at 28 °C in a 5% CO_2_ atmosphere in RPMI Dutch modified (Gibco, Invitrogen corporation, UK) cell culture medium buffered with 20 mM HEPES (Flow) and supplemented with 10% fetal calf serum (Sigma, St. Louis, USA), 1 mM piruvate, 2 mM glutamine, 50 µg/ml of gentamicin and 2.5 µg/ml of fungizone.

For infecting zebrafish, the SVCV isolate 56/70 [32] was grown in the ZF4 cell line at 22 °C by using the same cell culture media mentioned above except for 2% fetal calf serum. Supernatants from SVCV infected ZF4 cell monolayers were clarified by centrifugation at 4000 x g during 30 min and kept in aliquots at -70 °C. The SVCV titer was assayed by methylcellulose plaque assays [1]. Briefly, different dilutions of SVCV were used to infect ZF4 cell monolayers in 24-well plates for 1.5 hours. Then, the media were removed and each well covered with a solution of 2% methyl cellulose (SIGMA, St. Louis, US) in the infection medium. Plates were incubated at 22 °C for 5 days. The media with methyl cellulose was then removed and wells stained with crystal violet-formalin to count plaque forming units (pfu). To estimate SVCV titers in infected zebrafish, pooled head kidney and spleen from 4 fish were resuspended in 3 ml of RPMI cell culture medium prepared as described above, disrupted using a sterile nylon cell strainer (BD Falcon, MA, US) and a pestle and subsequently passed through 0.2 µm sterile filters to remove bacterial contamination.

### Infection of zebrafish with SVCV, tissue harvest and RNA extraction

Adult zebrafish of 2-3 g (~ 4 cm in length, XL size) were obtained from a local fish pet shop to study a situation that more closely resembles the variability of natural populations. Zebrafish were maintained at 24-28 °C in 30 l aquaria with tap-dechlorinated carbon-filtered water with 1 g of CaCl_2,_ 1 g of NaHCO_3_ and 0.5 g of Instant Ocean sea salts added to water resulting in a conductivity of 200-300 µS and pH of 7.8-8.2. The aquaria were provided with biological filters and fish fed with a commercial feed diet. For each infection experiment, groups of adult zebrafish were moved to 2 l aquaria provided with biological filters. To infect zebrafish with SVCV, cell culture medium containing 5-9 x 10^7^ pfu/ml was added to 2 l water tanks and fish exposed to the virus for 90 minutes. As stablished in parallel experiments, dosages of 7.1 x10^7^ pfu/ml killed 50% of the fish (LD_50_). Mock-infected zebrafish were incubated with cell culture medium in parallel experiments. Fish were then separated into infected and non-infected groups. We selected 2 day post infection for the first time point harvest because according to previous reports it is a time when the major percentage of genes are differentially transcribed [19,20,22-24,27-30]. In our infection conditions it was also the day before the first infection symptoms did appeared (data not shown). We selected 30 days post infection to harvest survivor fish because it was a time when mortalities had completely stopped and no signs of SVCV infection nor SVCV could be recovered from their head kidney and spleens (not shown).

After 2-days zebrafish were killed by an overdosage of anesthetics (methanesulfonate 3-aminobenzoic acid ethyl ester, MSS2) and used for the experiments. From 2 to 30 days, infected and non-infected fish were monitored 2-4 times daily and those with external hemorrhages killed by an overdosage of MSS2 to minimize their suffering. After 30-days, the survivor fish were killed by an overdosage of MSS2 as mentioned above and used for the experiments. Head kidney and spleen from fish 2- and 30-days after infection were harvested and pooled. Animals were handled in accordance with the National and European guidelines and regulations on laboratory animals care. Animal work was approved by the INIA corresponding Ethic Committee (authorization CEEA 2011/022).

For comparison of transcript expressions, non-infected and 2- or 30-days after SVCV infection zebrafish groups (n=6 zebrafish per group) were made. In each group, to isolate RNA for microarray hybridization, head kidney and spleen from each zebrafish were pooled to obtain enough RNA. The experiments were repeated 3 times for non-infected and 2-days infected groups and 2 times for 30-day survivors. The pooled head kidney and spleen were immediately immersed in RNAlater (Ambion, Austin, USA) at 4 °C overnight before being frozen at -70 °C until processed. RNA was extracted from sonicated (1 min x 3 times at 40 W in ice) zebrafish head kidney and spleen by following RNA isolation kit manufacturer instructions (RNeasy kits, Qiagen, Hilden, Germany). RNA concentrations were estimated by nanodrop and the presence of 18 and 28 S bands confirmed by denatured RNA agar electrophoresis (Sigma, Che.Co, MS, USA).

### Design of KEGG pathway-targeted microarrays

To design a zebrafish (*Danio rerio*, dre) microarray targeted to genes from the KEGG immune/infection pathways, we selected potentially-relevant human (*Homo sapiens*, hsa) pathways from the Kyoto Encyclopedia of Genes and Genomes (KEGG) database (http://www.genome.ad.jp/kegg/) accessed in February-March of 2012. The hsa pathways were selected to extract the corresponding dre gene orthologous because hsa were the most complete and not all the pathways were converted to dre in the KEGG web. The KEGG hsa pathways selected for study were grouped in: i) Growth and differentiation of immune response cells (“Hematopoietic cell lineage”, “Antigen processing and presentation” and “Natural killer cell mediated cytotoxicity”), ii) Inter-cellular response signals (“Complement and coagulation cascades”, “Chemokine signaling pathway”, “TGF-beta signaling pathway” and “Apoptosis”), iii) Intra-cellular response signals (B- and T-cell receptor, Toll-like receptor, MAPK, JAK-STAT, NOD-like receptor and RIG-I-like signaling pathways), iv) Mammalian pathways with unknown fish equivalents (“Fc gamma R-mediated phagocytosis”, “Fc epsilon RI signaling pathway” and “Intestinal immune network for IgA production”), and v) Human RNA viral infections (“Hepatitis C”, “Influenza A” and “Measles”).

Orthologous zebrafish mRNA short names (*italics*) were searched and retrieved from each human pathway box gene in http://www.kegg.jp/ssdb-bin/ssdb_best?org_gene=hsa and http://www.genome.jp/dbget-bin/www_bget?dre, to get the corresponding zebrafish accession numbers in http://www.genome.jp/dbget-bin/get_linkdb?-t+10+dre. When existing, each box gene of the KEGG hsa pathway was filled with the short name corresponding to the zebrafish orthologous. An effort was made to correct for ambiguously determined zebrafish orthologous short names by existing sequences, such as non existent short name, inappropriate accession number, etc. In those doubtful cases the short name used for human genes was followed by ? . 

To increase significance of the data, most of the genes were represented by several probes corresponding to different accession numbers. Because multipath genes were present in more than one pathway, those genes were replicated an average of ~40 times across the microarray.

To complete the KEGG pathway genes, we also included in the targeted microarray other immune-related genes retrieved by using keywords directly from the GenBank database of zebrafish mRNAs (http://www.ncbi.nlm.nih.gov/) accessed in February-March of 2012. We searched for the mRNAs corresponding to the following immune-related keywords: transcription factor, interferon, chemokine, interleukin, cytokine, defensin, macrophage, lymphocyte, antimicrobial, neutrophil, leukocyte, cytotoxic, natural killer, antiviral, antibacterial, LPS, Vig, antigen, cd, antigen, histocompatibility, phagocyte, viral, Mx, complement, immunoglobulin (Ig), hepcidin, IgG, IgM, Toll, T cell, B cell, dendritic, presenting, TANK, GNB, HMGB, TNF, and MHC. Retrieved sequences were filtered for duplicated accession numbers and non-related genes reviewed and eliminated manually. The corresponding gene sequences were then classified into 25 classes. Because of this hybrid selection strategy of both pathway and keyword sections, the targeted microarray offered a complementary coverage of possibilities for functional analysis.

Oligo probes of 60-mer and melting temperature (Tm) of 80 ± 3 °C were then designed for each of the above mentioned sequences by using the Array Designer 4.3 program (Premier Biosoft, Palo Alto CA, USA) and the zebrafish 56362 mRNA GenBank database (accessed in April, 2012). A total of 5212 probes in the pathway section of the targeted microarray design corresponded to 2286 accession numbers, since most of the genes had several accession numbers and genes were repeated in different pathways. These probes covered an average of 72.4% of the human genes present in the corresponding KEGG pathway (41.5-87.5% with 25 to 132 gene boxes per pathway, depending on the pathway). On the other hand, 6350 probes (accession numbers) retrieved with the use of keywords were obtained for the keyword section of the targeted microarray. Therefore, the final design contained 11562 annotated probes and 8636 accession numbers. *In silico* validation of the design was made by using BLAST of an statistically significant sampling of the probes to corroborate gene identification. The list corresponding to their 60-mer oligo probes was submitted to Agilent’s microarray design tool (https://earray.chem.agilent.com/earray/search.do?search¼arrayDesign). The pathway-targeted microarray in the 8x15K format was called zfin and corresponded to Agilent’s ID041401. A complete list of their gene IDs and gene names was also included in the Gene Expression Omnibus (GEO) platform with the submission number GPL15747.

### Quantitation of zebrafish transcripts after hybridization to the targeted microarray

Slides with the 8x15K format zebrafish 60-mer oligo microarray (zfin ID041401) were obtained from Agilent (http://www.chem.agilent.com/en-US/Products/Instruments/dnamicroarrays/zebrafisholigomicroarraykit/pages/gp58618.aspx). RNAs from head kidney and spleen from non-infected, infected (2-days) and survivor (30-days) zebrafish were kept frozen at -80 °C until all the experiments were hybridized and processed simultaneously. Labeling of 2 µg of RNA (~ 50 µg/ml) and hybridization to the microarrays were performed by NIMGENETICS (Cantoblanco, Madrid, Spain), complying with the Minimum Information About a Microarray Experiment (MIAME) standards. Briefly, high quality RNA were labeled with Cy3 (Amersham Pharmacia) by using SuperScript III reverse transcriptase (InVitroGen) and oligo(dT) primer, and the resulting cDNA was purified with Microcon YM30 (Millipore). The slides were pre-treated with 1% BSA, fraction V, 5 x SSC, 0.1% SDS (30 min at 50 °C) and washed with 2 x SSC (3 min) and 0.2 x SSC (3 min) and hybridized overnight in cocktail containing 1.3 x Denhardt’s, 3 x SSC 0.3% SDS, 2.1 μg/μl polyadenylate and 1 μg/μl yeast tRNA. Fluorescent signals were captured, processed and segmented using an Agilent scanner (G2565B, AgilentTechnologies) using the Agilent Feature ExtractionSoftware (v9.5) with the protocol GE1-v5_95, extended dynamic range and preprocessing by the Agilent feature extraction. Raw and normalized data were deposited in the GEO bank at http://www.ncbi.nlm.nih.gov/geo/query/acc.cgi?acc=GSE42263.

The gProccesedSignal was chosen for statistical analysis with the use of a home-made program developed in Origin pro vs 8.6 (Northampton, USA) by using their LabTalk programming language. The Origin graphic-analysis tool file carried out a first normalization step using the sum of all fluorescences within each data column corresponding to each microarray experiment. Normalized non-infected (control) outliers defined as fluorescence values outside their mean ± standard deviation per each gene were first masked from additional calculations. Control normalized outlier-free values were averaged to calculate their outlier-corrected mean for each gene (control mean). Folds were then calculated by applying the following formula for each gene, experimental value for each experiment / control mean. Fold outliers were then eliminated following the same criteria as above to calculate their outlier-free mean and standard deviation for each gene. Results obtained from each of the gene probes present in the microarray were then averaged and outliers eliminated. The Student t independent two-tail statistic associated p for each gene was finally computed from experimental outlier-free values compared to control outlier-free values. Genes with differential expression SVCV-infected /non-infected fold values > 1.5 (equal to the 30-day fold mean) or < 0.66 were defined as up or downregulated, respectively. Those transcript values which deviated from the null hypothesis using the one sample 2-tail independent t-test at p<0.05 were considered significatively expressed. A graphic representation of each of the zebrafish genes was drawn in KEGG-like pathways by using the Origin program. All the gene boxes appearing in the Origin graphs (short gene names in *italics*) corresponded to gene probes assayed in the experiments. To allow for a rapid graphic inspection of the main differentially expressed genes, up or downregulated gene *italic* letters were colored as follows: red <0.5, orange <0.66, dark green >1.5 and bright green >2 folds.

### Validation of the differential expression of multipath genes by reverse transcriptase and quantitative polymerase chain reaction (RTqPCR)

Microarray analysis results of the multipath genes were validated by RTqPCR using accession numbers of selected genes from the microarray to search for suitable primers with the Array Designer 4.3 program (Premier Biosoft, Palo Alto CA, USA). The list of genes contains 16 differentially expressed multipath genes corresponding to Table 1, a non-differentially expressed *mapk10* multipath gene and the *rplp0* normalizer gene [22]. Forward and reverse primers amplifying 100-120 bp were designed (Table S1). Immediately after RNA extraction, 10 μg of RNA from head kidney and spleen were converted to cDNA using the PrimeScript RT reagent kit (Takara, Japan) by 15 min at 37 °C and 15 sec at 85 °C and kept frozen at -70 °C until used. The resulting cDNA (25 ng cDNA per sample) was mixed with Power SYBR green PCR Master Mix (Applied Biosystems) in 15 μl of volume, heat denatured by 95 °C 10 min and amplified by 40 cycles of 95 °C 15 s and 60 °C 1 min in a LineGene 9600 Real-Time PCR system (Bioer Technology Co, Bingjiang, China). Samples were PCR amplified in 2 different amplification experiments and the average Ct used for the calculations. The relative number of molecules were calculated from the cycle threshold (Ct) data by using the 2^-delta^ relative quantitation method. Raw Ct were then normalized for each experiment by using the *rplp0* gene [22]. Outliers (values > or < means ± standard deviation) were identified and eliminated from the calculations by a home-made program in Origin 8.5. Fold for each gene were then calculated by the formula, relative number of molecules from infected or survivor fish / mean of relative number of molecules of non-infected fish. Means and standard deviations were then calculated for 2- and 30-day samples (n=3).

**Table 1:** List of differentially expressed genes which were present in at least 6 pathways ranked by the number of pathways.

**zebrafish**	**human**								
**short**	**short**	**accession**		**n°**	**2-**		**30-**		
**name**	**name**	**number**	**gene description**	**paths**	**day**	**±sd**	**day**	**±sd**	**n**
***tnfa***	*tnfa*	NM_212859	Tumor necrosis factor alpha	13	**0.8**	0.13	1.65*	0.42	68
***akt3a***	*akt3*	NM_001197201	~v-AK murine thymoma viral oncogene	12	0.55*	0.02	**0.73**	0.03	25
***pik3r5***	*pik3r5*	XM_002662345	phosphoinositide-3-kinase regulator subunit 5	12	**0.62**	0.58	**1.32**	0.1	41
***nfkb2***	*nfkb2* ^*a*^	NM_001001840	Nuclear factor kappa light polypeptide enhancer	11	**0.73**	0.06	2.01*	0.08	104
***chuk***	*chuk*	NM_200317	Conserved helix-loop-helix ubiquitous kinase	10	**1.25**	0.08	2.09*	0.14	44
***nfkbiab***	*nfkbia*	NM_199629	Nuclear factor kappa light polypeptide inhibitor	10	0.5*	0.01	**1.03**	0.02	63
***map2k1***	*map2k1*	NM_213419	Mitogen-activated protein kinase kinase 1	9	0.57*	0.16	**0.91**	0.13	35
***mapk14a***	*mapk14*	NM_131722	Mitogen-activated protein kinase 14a	8	0.56*	0.02	2.09*	0.05	49
***il1b***	*il1b*	NM_212844	Interleukin 1beta	7	0.58*	0.09	2.35*	0.12	43
***rac1***	*rac1*	NM_199771	Ras-related C3 botulinum toxin substrate 1	7	**0.73**	0.03	2.68*	0.11	60
***raf1b***	*raf1*	NM_001199755	~v-RAF-1 murine leukemia viral oncogene	7	0.58*	0.03	**0.96**	0.03	13
***ifnphi3***	*ifna/b*	NM_001111083	Interferon phi 3	6	1.9*	0.32	**0.75**	0.07	21
***ifng1-2***	*ifng*	NM_212864	Interferon, gamma 1-2	6	**1.09**	0.35	2.59*	0.39	17
***hras***	*hras*	XM_001923404	GTPase HRas-like	6	0.64*	0.01	**1.34**	0.13	6
***map3k7***	*map3k7*	NM_001020750	Mitogen activated protein kinase kinase kinase	6	**1.14**	0.24	1.73*	0.23	27
***traf6***	*traf6*	NM_001044752	TNF receptor-associated factor 6	6	**0.86**	0.07	1.59*	0.09	39

Differentially expressed (folds >1.5 or <0.66) genes and present in >6 KEGG pathways (multipath genes) together with their corresponding mean folds were filtered/extracted from the microarray data. The differentially expressed multipath genes were tabulated together with their corresponding mean folds (bold) and standard deviations at 2- and 30-days. The genes were then ordered by the number of pathways present per gene. Other genes common to >6 pathways were not differentially expressed, such as *mapk1* (NM_182888, common to 12 pathways), *sos2* (XM_685079, 9), *grb2* (NM_213035, 8), *mapk10* (NM_001037701, 7), *ikbkb* (NM_001123265, 7), *ikbkg* (NM_001014344, 6), *stat1a* (NM_131480, 6), and *vav3* (NM_001126393, 6). ^a^ According to the KEGG data, in hsa pathways the *nfkb* complexes were simplified to *nfkb1* (which would include *nfkb2*, *rela*, etc), however the *nfkb1* orthologous were identified as *nfkb2* in the zebrafish pathways that were available. * significatively <0.66 or >1.5-fold at the p<0.05 level. **n**, number of probes per gene.

### Promoter extraction and computational prediction of putative transcription factor binding sites (TFBS) and their corresponding transcriptional factors (TF)

To test for the possibility that genes showing up or downregulation across many pathways might have different regulation, we searched for different common TF among the multipath genes which might be differentiating upregulated from downregulated main responses. Therefore, the MatInspector program of the Genomatix software suite (http://www.genomatix.de) [33] was used to retrieve the zebrafish promoter sequences of the multipath genes to search for the presence of common transcription factor binding sites (TFBS), following a previously described similar strategy [34]. According to Genomatix, the zebrafish promoter sequences were extracted from the comparative genomic section of ElDorado (http://www.genomatix.de/online_help/help_eldorado/comparative_genomics_help.html) with an optimized length of 500 bp upstream of the first transcription start site (TSS) and 100 bp downstream of the last TSSs. The procedure identified many potential TFBS in the multipath gene promoters but only those with p<0.001 were considered for further study. The gene names of the TF corresponding to the identified TFBS were then retrieved from the MatBase (Genomatix). Finally, the differential expressions of each of the TF genes after 2- and 30-days of infection were extracted from the experimental data corresponding to the TF gene list which included 3104 unique TF gene probes in the keyword section of the targeted microarray. Because V$HOXC, V$FKHD and V$DMRT transcription binding sites were present in several related genes (at least in 4, 15 and 3 related genes, respectively) we used the most complete name of the genes but included only those genes of the family showing differential expression in the calculations. There were names of the genes differing in final a, b, c, 1, 2, etc, which normally refer to alleles on different chromosomes and/or sequence variants that have been considered the same genes for the calculations in a first approximation.

### Localization of *nfkb* by staining SVCV infected ZF4 cell monolayers with crossreacting antibodies

To study the presence and localization of the zebrafish *nfkb* protein after SVCV infection, we used anti-human *nfkb*/p65 (Thermo Fisher Scientific Inc., catalog number RB-1638-P). This polyclonal antibody was raised in rabbits to the C-terminal peptide of the human *nfkb* protein. Before being used, it was tested in parallel in ZF4 cells and HaCat (human) cells stimulated by poly I:C and LPS. The same pattern of expression and same translocation upon stimulation were observed in both cell lines. In addition, this antibody has been used in PAC-2 cells (another zebrafish fibroblast cell line similar to ZF4) prior to the study presented here [35]. To perform the test, ZF4 cell monolayers grown in 96-well plates at 24 °C were infected with SVCV (0.001 virus per cell). Two days later, the monolayers were fixed for 15 min in cold methanol. For *nfkb* detection, fixed ZF4 cell monolayers were incubated overnight with a polyclonal antibody anti-human *nfkb*/p65 (Thermo Fisher Scientific Inc.) diluted 200-fold in phosphate buffered saline (PBS) with 0.3% Triton X100 (Merck, Darmstadt, Germany) at 4 °C. The cells were then washed with PBS and incubated for 2 h with TRIC-labeled goat anti-rabbit IgG antibody (Sigma) diluted 300-fold in PBS with 0.3% Triton X100. Finally, the cells were washed 3 times with PBS. The monolayers were then counterstained with 4'-6-Diamidino-2-phenylindole (DAPI) during 20 min and washed. Stained cells were viewed at different wavelengths and photographed with an inverted fluorescence microscope (Nikon Eclipse TE2000-U; Nikon Instruments, Inc., NY) provided with a digital camera (Nikon DS-1QM). 

## Results

### Comparison between pathway-targeted and genome-wide microarrays

**Figure 1:**
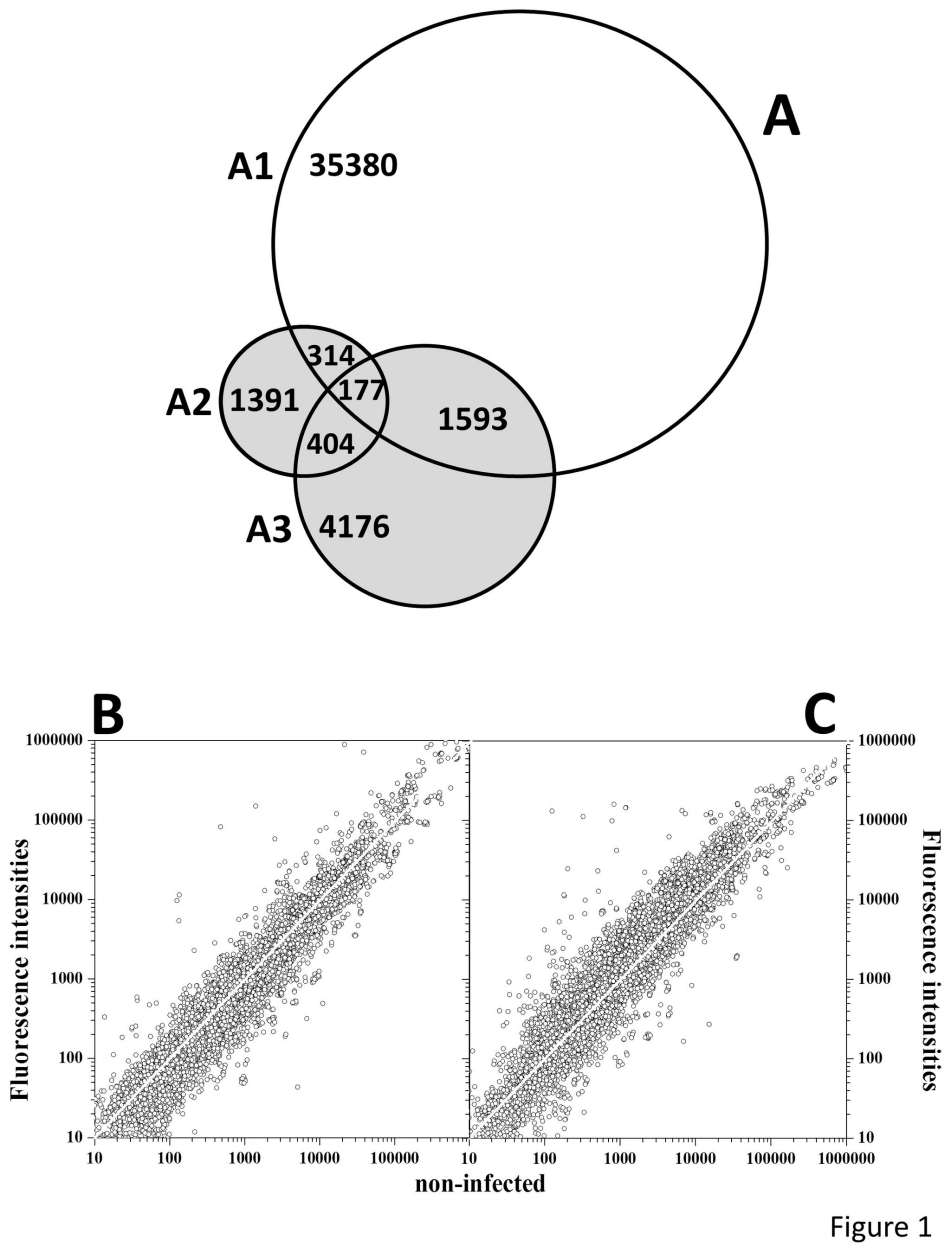
VENN diagram between genome-wide and targeted microarrays and comparison of fluorescence intensities obtained by hybridization to targeted microarrays of transcripts from 2- and 30-days after SVCV infection with those obtained from non-infected zebrafish controls. **A**) A VENN diagram was constructed by comparing unique accession numbers between genome-wide zebrafish vs2 ID019161 microarray of Agilent (43803 probes, 37464 accession numbers) and targeted microarray zfin ID041401 (11586 probes, 8636 unique accession numbers: 2286 and 6350 pathway and keyword sections, respectively). The online software from BioInfoRx (http://apps.bioinforx.com) was used. **A1**, wide-genome zebrafish vs 2 ID019161. **A2**, pathway section of the targeted microarray zfin ID041401. A3, keyword section of the targeted microarray zfin ID041401. **B**, **C**) The zfin ID041401 was used to estimate transcript levels in pooled head kidney and spleen from SVCV-infected and non-infected zebrafish after 2-days (**B**) or 30-days (**C**). The Figure shows the range of mean fluorescences obtained from different experiments (6 fish per experiment, n=3 for non-infected zebrafish and for SVCV infected zebrafish after 2-days and n=2 for SVCV infected zebrafish after 30-days). A white straight line shows fold = 1.

Comparison of our pathway-targeted microarray (11562 probes, 8636 accession numbers) with the genome-wide Agilent’s ID019161 (43803 probes, 37464 accession numbers), showed 5971 accession numbers to be unique to our pathway-targeted microarray (Figure 1A, VENN diagram), which means a 3-4 enrichment in immune-related probes. This enrichment has allowed a detailed analysis of the immune responses after SVCV infection. Furthermore, while in the pathway-targeted microarray all the probes were annotated, in the genome-wide microarray only 51.3% of the probes were annotated (according to manual estimations 7292 probes were labeled as unknowns, 8122 started with “zgc:”, “wu:”, “si:” or “im:” and 5895 used accession numbers as gene symbols).

### Infection of zebrafish with SVCV

Exposure of zebrafish to a high dosage of SVCV (5-9 x 10^7^ pfu/ml) during 90 min were used to minimize the possibilities of any zebrafish not being infected with the virus. Under these conditions, there were no infection symptoms nor mortalities after 2-days in any of the experiments, however beginning with 3-days, 80-90% of the exposed zebrafish showed external hemorrhagic symptoms in mouth, gills, lateral skin or fin bases 5-7 days later. At this time, SVCV levels in pooled head kidney and spleen ranged from 0.8 to 5 x 10^4^ pfu of SVCV per zebrafish (n=3 pools of 4 fish per pool). Mortalities after 7 days were of 38-46%, depending on the experiment (data not shown). Because mortalities occurred only during the first 15-20 days after infection, the 30-40% of alive zebrafish remaining after 30-days were considered survivors of the disease by being capable of overcoming the SVCV infection.

### Overview of the results of hybridization of samples to pathway-targeted microarrays

The RNA samples from SVCV infected zebrafish head kidney and spleen were hybridized to the pathway-targeted microarrays and fluorescence signals from non-infected, 2-day exposed and 30-day survivor normalized (data deposited in GSE42263). The normalized hybridization intensities ranged from 1 to ~ 1000000 fluorescence units. In samples from zebrafish after 2-days, 29.4% of the probes showed folds >1, while in samples from survivors, 56.9% of the probes showed folds >1 (Figure 1B and C).

### Effects of the SVCV infection in the pathways controlling host cells implicated in immune responses

The selected KEGG gene pathways having the highest influence in host cells implicated in immune responses were those involved in “hematopoietic cell lineage”, “antigen processing and presentation” and “natural killer cell mediated cytotoxicity” (Figure S1 in File S1).

Many of the stem, lymphoid (B- and T-cells) and myeloid (mast, basophil, eosinophil, dendritic/macrophage, neutrophil, erythroid and platelets) cell lineage markers and cytokines associated with their differentiation were downregulated after 2-days in contrast to their upregulation in survivors (not shown).

Antigen presentation through major histocompatibility complex (mhc) used by infected host cells to activate T-cell responses, were downregulated after 2-days (*cd8+* T-cytotoxic and NK cells and *cd4+* T-helper cells). In survivors, genes implicated in the activation of *cd8+* or cd4+ cells such as interferon gamma (*ifng1-2*), *cd8a* or *ifi30, creb1a*, were upregulated (not shown).

After 2-days, downregulation of genes required for natural killer (NK) cell activation such as *mhc1*, phosphoinositide-3-kinase (*pik3r5*), mitogen-activated protein kinase (*map2k1*), GTPase HRas-like (*hras*), fas ligand (*faslg*), *prf1* or caspase 3 (*casp3b*), suggested the inhibition of apoptosis and lysing of infected cells therefore favoring infection (Figure S1, A in File S1). Type I interferon *ifnphi3* (inducing a rapid and transient expression of many antiviral genes) [36] was upregulated. In contrast in survivors, the upregulation of tumor necrosis factor (*tnfa*), interferon gamma (*ifng1-2*) (inducing longer-time responses than *ifnphi3*) [37-39], ras-related C3 botulinum toxin substrate (*rac1*) and *gzmb* suggested the activation of NK cells (Figure S1, B in File S1).

### Effects of the SVCV infection in the pathways controlling inter-cellular signaling

The signaling pathways involved in the complement and coagulation cascades, Chemokine, TGF-beta and apoptosis (Figure S2 in File S1), have the highest influence in controlling inter-cellular signaling.

After 2-days, the coagulation cascade was reduced because of upregulated inhibiting serpins (*serpind1*, *serpinc1*), favoring SVCV-dependent host hemorrhages. On the other hand, complement pathways were activated because of the downregulation of inhibitors (*cr2, cd59*) while the lectin and antigen-antibody complement pathway (*hbl4*, *masp2*, *c* 2) and bradykinin vasoactive peptides (*kng1*) were upregulated, favoring cell lysis, chemotaxis and inflammation, respectively (not shown). Nevertheless, the complement component 1 subcomponent q (*c1qa*) was downregulated, most probably to reduce the consequences of viral antigen recognition by possible preexisting host anti-SVCV antibodies. In survivors the coagulation cascade still appears inhibited (upregulation of *serpind1* and downregulation of *f9b, f10, f2*) while the bradykinin-dependent inflammation (*kng1*) and the connections with the complement pathway (downregulation of *serpinc1*, *serpina1*, *a2m* inhibitors) were upregulated. There were few changes in the expression of the genes corresponding to the alternative, lectin and antigen-antibody complement paths in survivors (not shown).

Although most genes in the chemokine pathway were downregulated after 2-days (ie. *nfkbiab*, *hras, raf1b, map2k1*), downregulation of nuclear factor kappa light polypeptide inhibitor (*nfkbiab*) would favor nuclear translocation of pleiotropic anti-viral nuclear factor kappa light polypeptide gene enhancer B-cell 2 (*nfkb2*). In survivors, most of the above mentioned chemotactic related genes were upregulated, possibly promoting cell migration towards head kidney and spleen. Therefore, at least some of the observed changes in gene expression could be due to changes in cell type abundance in head kidney and spleen instead of within a constant cell population.

Signaling by the transforming growth factor-beta (tgfb), also showed downregulation after 2-days while survivors showed downregulation of inhibitors (*chd*, *tsp4*) and upregulation of downstream genes (*ifng1-2, tnfa*).

After 2-days, most of the gene transcripts in the intrinsic apoptosis were unchanged while both in the survival (proinflammatory interleukin 1 beta *il1b, pik3r5, akt3a, nfkbiab*) and in the extrinsic apoptosis (*faslg, apaf1, casp3*) were downregulated (Figure S2, A in File S1). In contrast, survivors showed upregulation in many of the genes involved in the survival path (*tnfa*, *il1b*, *nfkb2, tradd*, *myd88*, *irak3*, *map3k14*, *birc2, bcl2*) (Figure S2, B in File S1). Because both *birc2* and *bcl2* were downregulators, their upregulation should inhibit apoptosis. On the other hand, genes participating in the intrinsic apoptosis pathway were also upregulated, most probably as a result of prolonged stress due to accumulation/removal of misfolded proteins after infection.

### Effects of SVCV infection in the pathways controlling intra-cellular signaling

**Figure 2:**
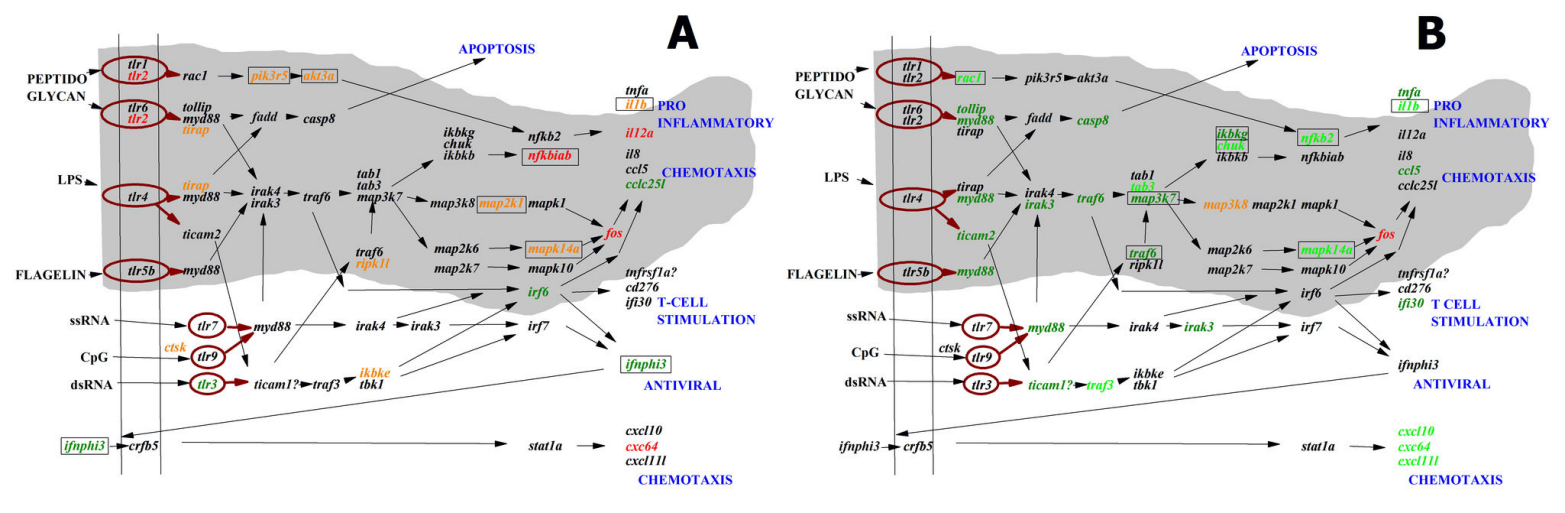
“Toll-like receptor signaling pathway” of zebrafish genes after SVCV infection. The gene boxes containing the zebrafish gene short names in *italics* and their relationships were obtained from the corresponding human KEGG-pathway. All those genes appearing in the Figure were assayed for transcript expression. Capital letters, inputs (black) and outputs (blue) of the pathway. Vertical parallel lines, cellular membranes. Continuous black arrows, activation between gene products. Discontinuous black arrows, inhibition between gene products. Gray form, main pathway. Folds were calculated as described in methods by the formula, fluorescence value 2- or 30-days after infection / fluorescence mean value in non-infected controls. Red *italic* letters, downregulated transcripts with folds <0.5. Orange *italic* letters, downregulated transcripts with folds <0.66. Bright green *italic* letters, upregulated transcripts with folds >2. Dark green *italic* letters, upregulated transcripts with folds >1.5. Black *italic* letters, not differentially expressed. Gene short names surrounded by an square, multipath genes. Gray form, downstream pathway beginning with cell membrane toll-like receptors (tlr1, tlr2, tlr4, tlr5b and tlr6). Ovoid brown forms, *tlrs*. Gene *italic* letters were colored as follows: red <0.5, orange <0.66, dark green >1.5 and bright green >2 folds. **A**, 2-days after SVCV infection. **B**, 30-days after SVCV infection (survivors).

Intra-cellular KEGG pathways describing downstream signaling after pathogen-derived or induced molecules are recognized by host cell receptors were B-cell (Figure S3 in File S1), T-cell (Figure S4 in File S1), Toll-like (Figure 2), MAPK (Figure S5 in File S1), JAK-STAT and RIG.

B-cell receptors, co-inhibitors or co-immunostimulators and many of their downstream genes (such as *pik3r5*, *akt3a*, *map2k1*, *nfkbiab, hras, raf1b, map2k1*) in the B-cell receptor signaling pathway were downregulated after 2-days (Figure S3, A in File S1), most probably causing inhibition of B-cell proliferation/ differentiation and therefore delaying antibody production. However, few of these genes were upregulated in survivors (*nfkb2, rac1, chuk*), suggesting that after 30-days antibody production was still reduced (Figure S3, B in File S1). All these data suggest that resistance to SVCV resulted mainly from innate responses.

T-cell downstream membrane co-inhibitors (*ctla4*, *ptpn6*) or co-immunostimulators (*pik3r5*) and downstream genes (*map2k1, akt3a*, *nfkbiab*) in the T-cell pathway were downregulated after 2-days (Figure S4, A in File S1), most probably to inhibit T-cell proliferation/differentiation. In contrast in survivors, downstream genes (*nfkb2, mapk14a, chuk*) and output (*ifng1-2, tnfa*) genes were upregulated (Figure S4, B in File S1). It is noteworthy that in both B- and T-cell pathways *fos* (v-fos FBJ murine osteosarcoma viral oncogene) was downregulated any time after infection. In mammalians, *fos* is a transcription factor (TF) which dimerizes with *jun* (whose expression remained unchanged any time after infection) to form the AP-1 TF complex, a general activator of proliferation and differentiation of many genes against infection and cellular damage. SVCV managed to maintain this important factor downregulated through the beginning of infection to survivors.

Toll-like receptors (*tlrs*) recognize pathogen associated molecular patterns (PAMPs) such as peptidoglycans, lipopeptides, flagellin, CpG motifs, single stranded RNA (ssRNA) and double stranded RNA (dsRNA) by membrane or cellular proteins to produce pro-inflammatory, chemotaxis, T-cell stimulation, interferons (*ifns*) and/or many other *ifn*-induced antiviral signals (Figure 2). Remarkably, the highest percentage (30.4%) of genes that were differentially expressed after 2-days belonged to this pathway. Thus downregulation of *tlr2* and many of their corresponding downstream genes (*pik3r5, akt3, nfkbiab, map2k1, mapk14a, tirap, fos*), confirmed the downregulation observed in many of their output pro-inflammatory signals (*il1b, il12a, cxc64*) after 2-days. Only upregulated *ifnphi3, tlr3, irf6*, and *cclc25l* bypassed the general downregulation downstream of *tlr* recognition (Figure 2, A). On the other hand, the percentage of genes that were differentially expressed in this pathway in survivors was also one of the highest (42.0%). In contrast to 2-days, upregulation of genes (*nfkb2, mapk14a, rac1, traf6, chuk, tollip, myd88, ticam2, irak3, casp8, traf3*) in survivors might increase proinflammatory and chemotaxis outputs such as *tnfa*, *il1b* and chemokines (*ccl5*, *cxcl10*, *cxc64, cxcl11*) (Figure 2, B).

Many genes implicated in the mitogen-activated protein kinase (MAPK) complex network were downregulated after 2-days (*map2k1*, *akt3a*, *mapk14a, fos*) including those corresponding to extracellular activating signals (*il1b*, *ngf, bdnf, ntf3, faslg*) and their receptors (*tlr2, agfra, fgfr1a, pdgfra*) (Figure S5, A in File S1). In survivors, the extracellular signals (*tnfa, il1b*) and many downstream genes (*nfkb2, chuk, rac1, mapk14a, map3k7, traf6*) were upregulated (Figure S5, B in File S1), suggesting that outputs of these paths such as proliferation, differentiation and inflammation signals were increased.

The JAK-STAT pathway was nearly unaffected during the course of SVCV infection (not shown). The RIG receptor pathway which possibly recognizes dsRNA intermediates of SVCV replication, showed downregulation (*nfkbiab, mapk14a*) and upregulation (*isg15, irf6*) leading to decreased *il12a* or increased *ifnphi3* after 2-days. In contrast in survivors many genes were upregulated (*nfkb2, chuk, mapk14a, traf3*, *tradd, tank*), leading to increased *tnfa* levels (not shown).

### Effects of SVCV infection in pathways not yet described for zebrafish

Because fish IgG-, IgE- or IgA have not been identified in fish, the human “Fc gamma R-mediated phagocytosis”, “Fc epsilon RI signaling" and “intestinal immune network for IgA production” pathways might not be present in zebrafish. Nevertheless in IgA for example, functional similarities with the IgZ genes implicated in the zebrafish mucosal responses could exist. The corresponding zebrafish orthologous pathways showed similar trends than most of those pathways described above. Briefly, most of the genes in each of the Fcg, Fce or IgA pathways mentioned above were downregulated after 2-days (for instance *pik3r5, akt3a, raf1b, map2k1* in Fcg, *pik3r5, akt3a, mapk14a, raf1a, hras, map2k1* in Fce and *cd40lg, cxcl12a, ccl1, ccl25b, il15* in IgA pathways), while other genes become upregulated in survivors (for instance *rac1* in Fcg, *rac1, mapk14a, tnfa* in Fce, and *mhc2, cxcl12a* in IgA) (not shown). These results suggest that there might be functional equivalents of those human pathways/genes in zebrafish although they have not been identified yet.

### Effects of the SVCV infection in human RNA viral infection pathways

The zebrafish pathways made up with genes orthologous to those of human RNA viral infections such as hepatitis C, influenza A or measles, confirmed that most of their genes were downregulated after 2-days while mostly different genes, become upregulated in survivors (not shown). Most important outputs after 2-days were upregulation of *ifnphi3* in hepatitis and upregulation of *ifnphi3* and *mxa* or downregulation of *il1b* and *il12a* in influenza and measles, respectively. Other downregulated genes after 2-days were *pik3r5, akt3a, nfkbiab, hras, mapk14a* in hepatitis, *pik3r5, akt3a, il1b, il12a, raf1b, map2k1* in influenza and *pik3r5, akt3a, nfkbiab, il1b, il12a* in measles. On the other hand, in survivors the most important outputs showed no upregulated genes in hepatitis, upregulation of *il1b* both in influenza and measles and *tnfa* upregulation in measles. Other upregulated genes in survivors were *mapk14a, nfkb2, chuk, traf6* in hepatitis, *mapk14a, tnfa, il1b* in influenza and *nfkb2, chuk, map3k7, traf6, il1b* in measles.

### Effects of the SVCV infection in zebrafish genes not included in any KEGG pathways

**Figure 3:**
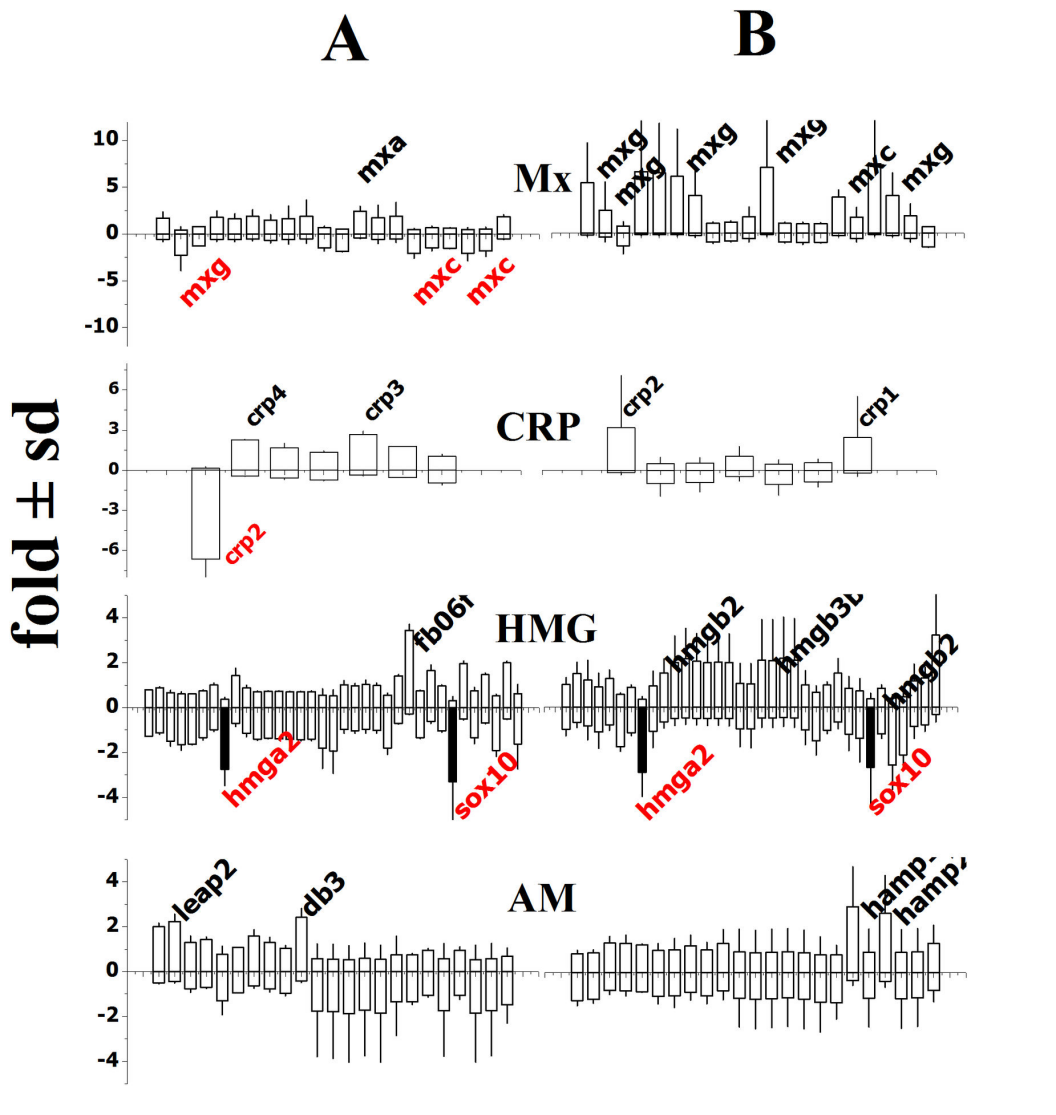
Differential expression of Mx, C-reactive proteins (CRP), high mobility group (HMG), and antimicrobial peptides (AM) 2- and 30- days after SVCV infection. The data were obtained from the keyword section of the targeted microarray. After normalization, means and their standard deviations were represented. Short gene names (*italics*) with differential expression folds > 2 (black names) or < 0.5 (red names) are to the right of the corresponding bars. The same short gene names were used for different bars because they corresponded to different probes per gene. Black bars, similar folds at 2- and 30-days. +, increased folds in black. -, decreased folds in red. **A**) 2-days after infection. **B**) 30-days after infection.

In order to study genes not yet included in any KEGG pathways but which might be important in SVCV infections, a complementary set of genes grouped in classes were also included into the microarray (see methods). Figure 3 shows the expression profiles in the Mx (Myxovirus-induced proteins), CRP (C-reactive proteins), HMG (high mobility group proteins), and AMP (antimicrobial peptides) gene classes. After 2-days, only the *mxa* transcripts were upregulated while *mxc* and *mxg* were downregulated. In contrast both *mxc* and *mxg* were upregulated in survivors (Figure 3, Mx). The different expression profiles corresponded to different isoform sequence groups (*mxa*+*mxb* and *mxc*+*mxe*+*mxg*), suggesting that Mx isoforms might have different functions in zebrafish. Similarly, after 2-days, only *crp3* and *crp4* transcripts were upregulated while *crp2* was downregulated. In contrast, both *crp1* and *crp2* were upregulated in survivors (Figure 3, CRP), suggesting different functions for CRP isoforms also because of their sequence grouping [40]. Finally, a similar phenomenon could be described for *hmg* genes, where *hmgb2* and *hmgb3b* were upregulated only in survivors (*hmgba2/sox10* levels remained constantly downregulated thus serving as internal controls that validate other results) (Figure 3, HMG). With respect to antimicrobial peptides (AM), *leap2* (liver expressed antimicrobial peptide) and *db3* (β defensin 3) were upregulated after 2-days while 2 isoforms of *hamp* (hepcidins) were upregulated in survivors (Figure 3, AM).

### Identification of differentially expressed genes common to several pathways

The numerous genes that were regulated and the identification of common genes in different pathways, suggested that those responses could be dependent of a few master genes. Master gene candidates could be amongst those present in multiple pathways (multipath genes), since their regulation through the complex network of genes would interconnect pathways and therefore they might have the widest impact on the outcome of the SVCV infection. To test for differentially expressed multipath genes, data was systematically searched for genes that: i) showed differential expression folds >1.5 or <0.66, ii) were significatively different (p<0.05) from one of the 1.5/0.66 thresholds in at least one of the 2- or 30-day data, and iii) were common to the highest number of pathways (>6 pathways).

Sixteen differentially expressed multipath genes filling the above mentioned criteria were identified (Table 1). Significatively downregulated multipath genes after 2-days were the following genes: v-AK murine thymoma viral oncogene (*akt3a*), nuclear factor of kappa light polypeptide gene enhancer in B-cells inhibitor alpha (*nfkbiab*), mitogen-activated protein kinase kinase 1 (*map2k1*), mitogen-activated protein kinase 14a (*mapk14a*), interleukin 1beta (*il1b*), v-RAF-1 murine leukemia viral oncogene (*raf1b*) and GTPase HRas-like (*hras*) while only type I interferon phi 3 (*ifnphi3*) was upregulated. Significatively upregulated multipath genes in survivors were: tumor necrosis factor alpha (tnfa), nuclear factor kappa light polypeptide gene enhancer in B-cells 2 (p49/p100) (*nfkb2*), conserved helix-loop-helix ubiquitous kinase (chuk), *mapk14a*, *il1b*, ras-related C3 botulinum toxin substrate 1 (*rac1*), interferon gamma 1-2 (*ifng1-2*), mitogen activated protein kinase kinase kinase (*map3k7*), and TNF receptor-associated factor 6 (*traf6*). Only *mapk14a* and *il1b* were both downregulated after 2-days and upregulated in survivors. Significant multipath genes common to > 10 pathways were the *tnfa*, *akt3a, nfkb2, chuk* and *nfkbiab* genes; all of them were present in the Toll-like signaling pathway except *tnfa* (Figure 2, rectangled boxes). Of all the above mentioned genes, only phosphoinositide-3-kinase regulator subunit 5 (*pik3r5*) showed non significant differential expressions at the p<0.05 level; nevertheless it was maintained in the multipath gene list owing to its close relationship to *akt3a*. In contrast, other multipath genes such as *mapk1* (common to 12 pathways), *sos2* (9), *grb2* (8), *mapk10* (7), *ikbkb* (7), *ikbkg* (6), *stat1a* (6) and *vav3* (6), were not differentially expressed at the p<0.05 level.

**Figure 4:**
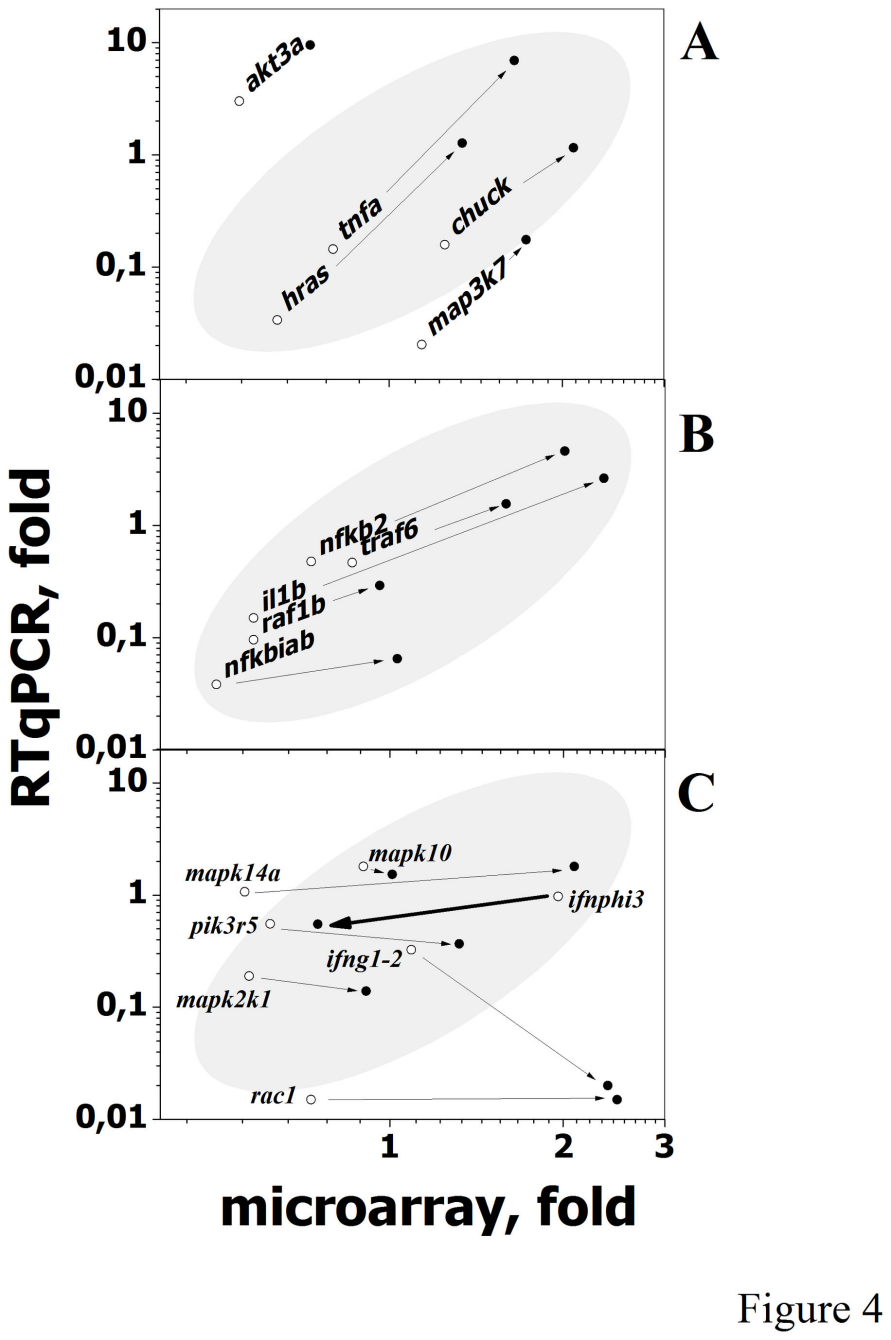
Microarray hybridization and RTqPCR fold comparison of differentially expressed multipath genes. Microarray folds of the differentially expressed multipath genes from Table 1 were compared with the corresponding folds obtained by RTqPCR as described in Methods. To increase clarity the genes were distributed in three groups: **A**, **B** and **C**. Points inside the shadowed ellipses include most of the data. Logarithmic scales were used to best compare all data because of the wide relative differences between microarray and RTqPCR fold values. ○, Mean folds from head kidney and spleen from 2-day infected zebrafish. ●, Mean folds from head kidney and spleen from 30-day survivor zebrafish. Arrows indicate the direction of the fold changes from 2- to 30-days by both microarray and RTqPCR estimations. The *mapk10*, a multipath gene whose fold did not changed from 2- to 30-days, was included as a control (Figure 4C).

To validate the folds of the differentially expressed multipath genes of Table 1 obtained by hybridization to the targeted microarrays, RTqPCR was performed on the same RNA samples by using different primers. Results showed that 79.4% of the microarray folds were proportional to their corresponding RTqPCR folds (Figure 4 A,B,C points inside the gray ellipse). On the other hand, increasing folds from 2- to 30-days were confirmed for *akt3a, tnfa, hras, chuck*, and *map3k7* (Figure 4A, higher slopes) and *nfkb2, traf6, il1b, raf1b* and *nfkbiab* (Figure 4B) but not for *mapk14a*, *pik3r5*, *map2k1, rac1, ifnphi3* (no changes) or *ifn1-2* (decreasing from 2- to 30-days) (Figure 4C). In addition, *mapk10*, a multipath gene chosen as a control because its folds did not changed from 2- to 30-days, was confirmed by RTqPCR (Figure 4C).

**Figure 5:**
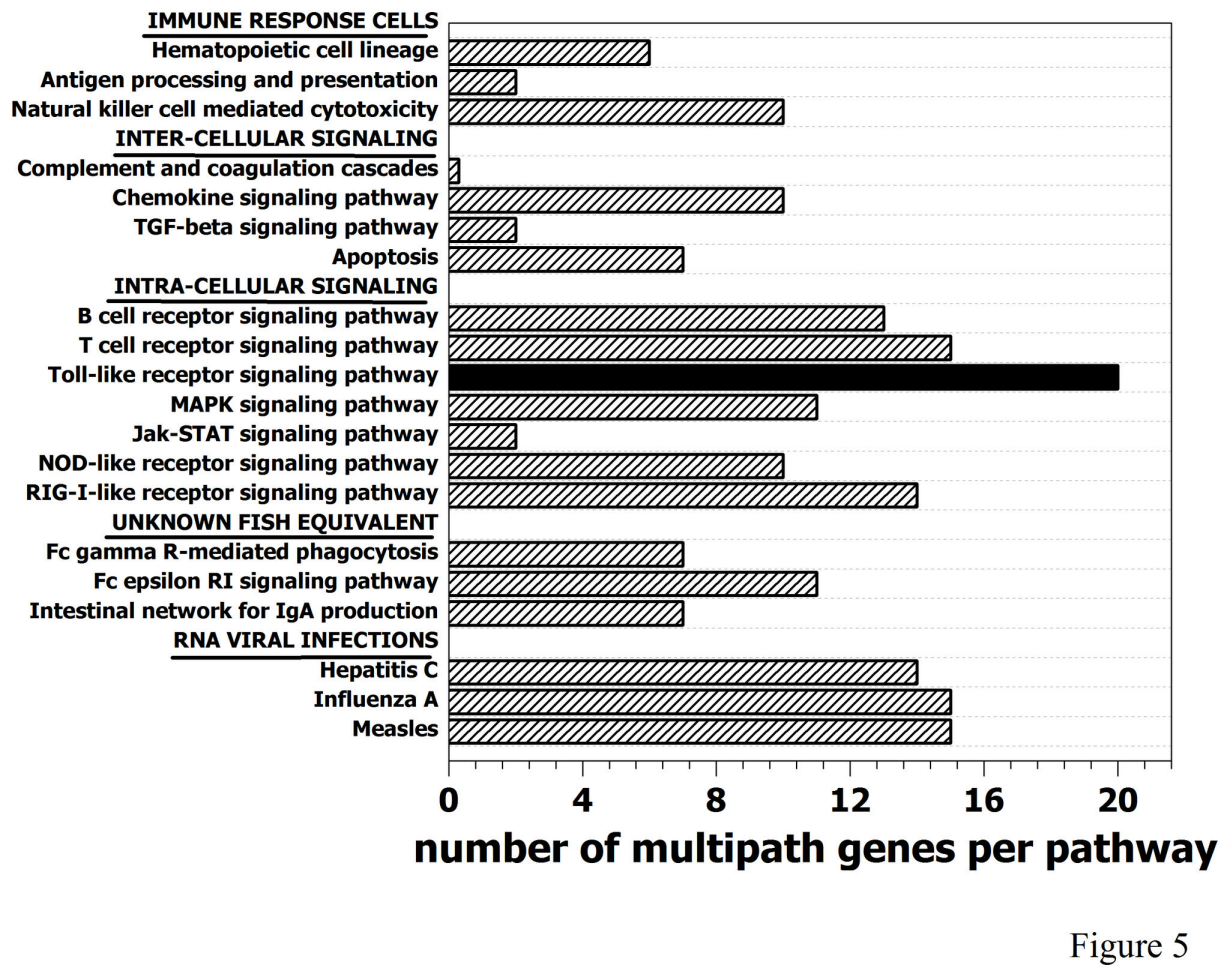
Number of multipath genes per KEGG pathway. The number of multipath genes having differential expression folds <0.66 or > 1.5 (differentially expressed multipath genes of Table 1) in each of the 20 studied KEGG pathways, were represented. Black bar, Toll-like receptor signaling pathway. Hatched bars, rest of the pathways.

The gene pathways containing the highest number of differentially expressed multipath genes were Toll-like, B-cell, T-cell and RIG receptors, and human-like RNA viral infections (hepatitis C, influenza A and measles) (Figure 5). In contrast, the pathways involved in the complement and coagulation cascade or TGF-beta and JAK-STAT signaling have a minimum of differentially expressed multipath genes (Figure 5). Among the differentially expressed multipath genes identified, the *nfkb2* seems one of the most important because it is upstream of many of the other identified multipath genes (*akt3a, pik3r5, chuk, nfkbiab, map2k1, mapk14a, rac1, map3k7, ikbkg, traf6*) and its downstream activities include proinflammatory multipath *tnfa* or *il1b*. The *nfkb2* was upregulated in survivors in 55% of the studied pathways (Toll-like, RIG-I-like, NOD-like, T cell and B cell signaling receptors, apoptosis, MAPK and chemokine signaling and hepatitis, influenza and measles infection pathways). The importance of all the multipath genes mentioned above relays in their participation together with *nfkb2* to target ~ 200 additional genes important for a wide variety of host responses to infection [41]. Consequently with this key position, *nfkb* have been described as a viral target for shutoff in mammalian viral infections such as herpes [42], poliovirus [43] and rhabdovirus [44]. Shutoff of *nfkb* has been also reported in salmonid birnavirus infections [45] and in zebrafish after activation of *tlr4* [46].

### Identification of putative transcription factors common to regulated multipath genes

Although in most pathways (except for the complement and coagulation cascade) many genes were downregulated after 2-days and upregulated in survivors, the regulated genes were not exactly the same. Since the genes in each of the down or upregulated group might be critical for either initiation of infection or survival, respectively, a group-dependent regulation could exist. To make a preliminary test of that hypothesis, the multipath genes of Table 1 were separated into downregulated (*pik3r5, nfkbiab, map2k1, mapk14a, il1b, raf1b, hras*) and upregulated (*tnfa, akt3a, nfkb2, chuk, mapk14a, il1b, rac1, ifnphi3, traf6, ifng1-2, map3k7*) groups and their promoters searched for common transcription factor binding sites (TFBS, V$TFBS) in each group. Promoter sequences could be retrieved by the MatInspector program (Genomatix) for all the multipath genes except for *akt3a*. The search identified many common TFBS for down and upregulated multipath gene groups (not shown). However, when the results were filtered by an strong significance level (p<0.001), the list was reduced and group-dependent TFBS identified. The TF corresponding to the identified TFBS were then retrieved by using the Matbase (Genomatix). Those TF showing differential expression after SVCV infection were *dmrt2a* in the downregulated group and *pou1f1, hoxa, hoxd, foxb1.2, foxd3, foxk* and *fox1* in the upregulated group (Table 2). Other retrieved TF were not differentially expressed. These results suggested that these TFs might be contributing to a differential control of transcription of the down and upregulated multipath gene groups.

**Table 2:** Predicted common transcription factor binding sites (V$ TFBS), presence of TFBS in multipath gene promoters, transcription factor (TF) and differential expression of TF genes after SVCV infection.

												**Differential expression of TF genes**				
**V$TFBS**	**Multipath gene promoters**										**TF**	**2-day mean**	**±sd**	**30-day mean**	**±sd**	**n**
**UP:**																
	***tnfa***	***nfkb2***	***chuk***	***mapk14a***	***il1b***	***rac1***	***ifnphi3***	***traf6***	***ifng1-2***	***map3k7***						
V$NKX1	**X**	**X**	**X**	**X**	**X**		**X**				*nkx1.2l*	**0.91**	**±**0.12	**1.03**	**±**0.19	4
V$PIT1	**X**	**X**	**X**	**X**	**X**	**X**	**X**			**X**	*pou1f1*	**0.59**	**±**0.12	2.88*	±0.49	2
V$HOXC	**X**	**X**	**X**	**X**	**X**	**X**	**X**	**X**	**X**	**X**	*hoxa*	0.31*	**±**0.01	**1.16**	±0.01	2
	**X**	**X**	**X**	**X**	**X**	**X**	**X**	**X**	**X**	**X**	*hoxd*	**0.56**	±0.44	2.66*	±0.01	4
V$EVI1	**X**	**X**	**X**	**X**	**X**	**X**	**X**	**X**	**X**	**X**	*bcl11a*	**1.15**	**±**0.55	**0.80**	±0.02	3
V$FKHD	**X**	**X**	**X**	**X**	**X**	**X**	**X**	**X**	**X**	**X**	*foxb1.2*	6.79*	±1.76	**0.75**	±0.22	3
	**X**	**X**	**X**	**X**	**X**	**X**	**X**	**X**	**X**	**X**	*foxd3*	**0.87**	±0.01	0.42*	±0.01	2
	**X**	**X**	**X**	**X**	**X**	**X**	**X**	**X**	**X**	**X**	*foxk*	2.15*	±0.50	**1.79**	±0.10	6
	**X**	**X**	**X**	**X**	**X**	**X**	**X**	**X**	**X**		*fox1*	**0.63**	±0.26	3.22*	±0.60	5
**DOWN:**																
	***pik3r5***	***nfkbiab***	***map2k1***	***mapk14a***	***il1b***	***raf1b***	***hras***									
V$PAXH	**X**	**X**		**X**	**X**		**X**				*pax6*	**0.68**	**±**0.33	**0.71**	**±**0.09	2
V$DMRT	**X**	**X**	**X**	**X**	**X**	**X**	**X**				*dmrt2a*	**0.50**	±0.03	1.97*	**±**0.04	3
V$OCT1	**X**	**X**	**X**	**X**	**X**	**X**	**X**				*pou2*	**0.62**	**±**0.18	**2.26**	±2.34	2

The differentially expressed multipath genes of Table 1 were separated into upregulated (UP) and downregulated (DOWN). Their promoters were then retrieved by using MatInspector (Genomatix software suite) and the presence of common transcription factor binding sites (**V$ TFBS**) in the promoters determined by using their "search several different sequences for common TF sites" feature. The *akt3a* promoter could not be retrieved by MatInspector. The V$ TFBS with p<0.001 were used to identify the corresponding transcription factor genes (TF) in the Matbase of Genomatix. Finally, the differential expressions of each of the TF genes after 2- and 30-days of infection were retrieved from the 3104 TF gene list included in the keyword section of the targeted microarray. * significatively <0.66 or >1.5-fold at the p<0.05 level. Because V$ HOXC, V$ FKHD and V$ DMRT were present in several related genes (at least 4, 15 and 3 related genes, respectively), genes were grouped and only those members of each family showing differential expression were represented in the Table to make it shorter. **n**, number of probes per gene. **X**, presence of the V$ TFBS in the corresponding multipath gene promoters. Empty, absence of the corresponding V$ TFBS.

### Location of *nfkb* protein in ZF4 cell monolayers infected with SVCV

**Figure 6:**
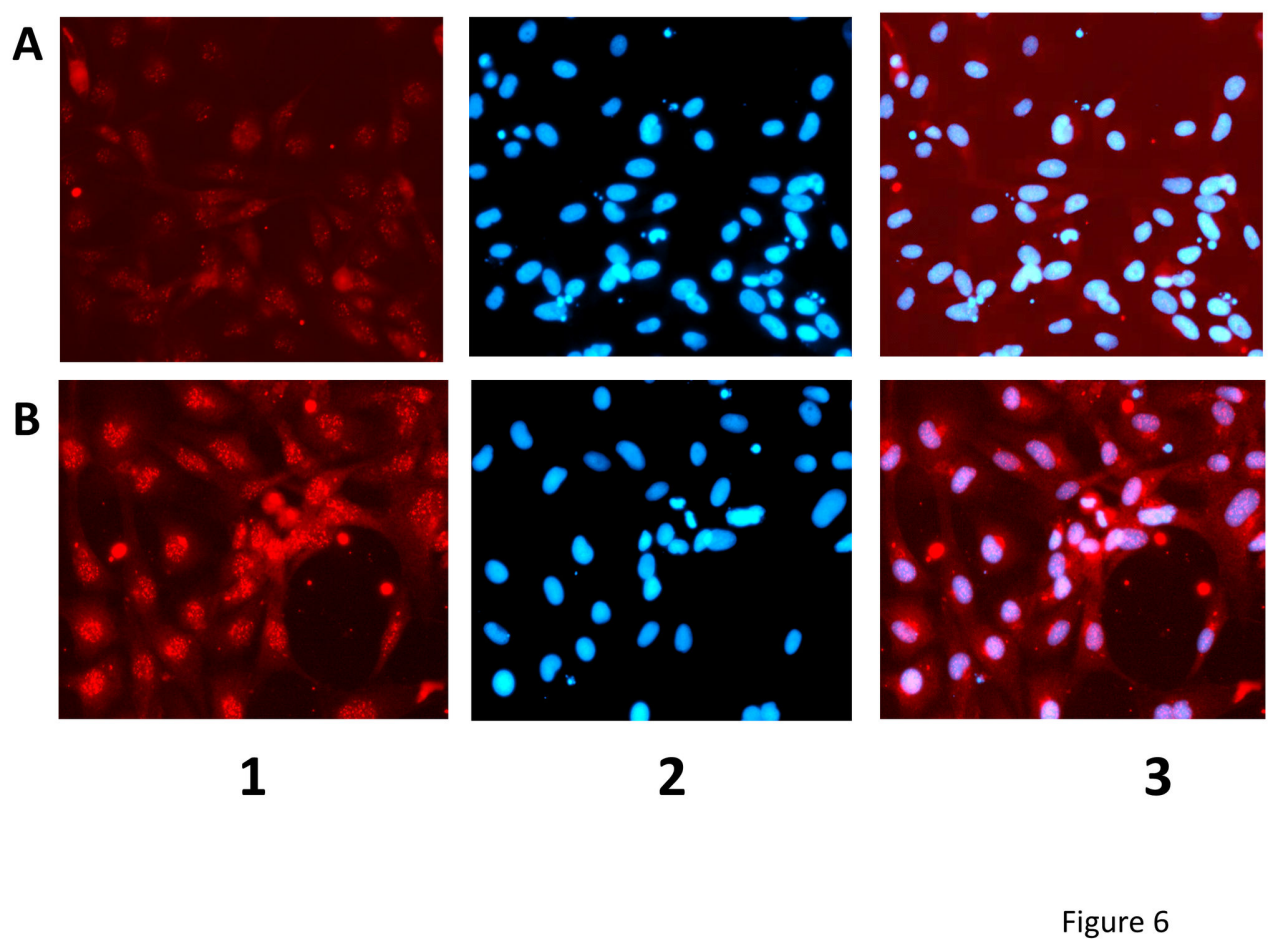
Immunofluorescence microscopy of ZF4 cell monolayers after SVCV infection reveals synthesis of *nfkb2* protein and its nuclear translocation. ZF4 cell monolayers were either mock infected (**A**) or infected with 0.001 SVCV per cell (**B**). Two days later the cell monolayers were stained with anti-human *nfkb/p65* -TRIC-labeled secondary antibodies and with DAPI (nuclear staining) as indicated in methods. **1**, Fluorescence with anti-*nfkb/p65* -TRIC- labeled antibodies. **2**, Fluorescence with DAPI. **3**, Merged fields 1 and 2.

To estimate changes in their subcellular localization and/or possible correlations between transcript expression and protein levels, we focused on the *nfkb* family of proteins since *nfkb2* was identified as one of the key targets for SVCV-induced responses and one of the more pleiotropic multipath genes of Table 1. Taking advantage of the existence of anti-human *nfkb/p65* antibodies crossreacting with the zebrafish nfkb complex, we further studied the possible involvement of complexes related to *nfkb* in SVCV infected ZF4 cells by immunostaining. The *nfkb* fluorescence was localized mainly in the cytoplasm of non-infected ZF4 cells as expected (Figure 6A). However, the fluorescence appeared also in the nuclei and its intensity increased in cytoplasmic granulae in SVCV-infected ZF4 cells, suggesting both induced translocation and new synthesis of zebrafish *nfkb* (Figure 6B). 

## Discussion

### Possible mechanisms of differentially expressed multipath genes and pathways

We have identified 16 differentially expressed SVCV-dependent genes common to > 30% of the pathways studied (here called multipath genes). The number of pathways could have been larger if more pathways from the KEGG or other databases (ie. Wikipaths, Nature Pathway Interaction Database, Pathway Commons, etc) had been included in the design. Work to enrich the pathway-targeted microarray approach with more pathways is ongoing.

By studying hypothetical zebrafish pathways with genes derived from their human orthologous, we are assuming that those are similar. Although it is a reasonable hypothesis, that might not be completely accurate. However, given the high amount of molecular data required to define gene pathways for each biological specie, this approach seems to be the simplest to progress in our understanding of fish responses. The possibility that the observed changes in gene expression could be due to changes in cell type abundance in head kidney and spleen due to cell migration rather than within a constant cell population must be also considered as a possible mechanism to explain the profiles of differential expression. Future histological analysis of kidney and spleen, might be used to demonstrate likely SVCV-effects on cell type abundance in head kidney and spleen.

Results showed that many genes were downregulated after 2-days (including multipath genes *pik3r5, nfkbiab, map2k1, mapk14a, il1b, raf1b, hras*) while many other genes were upregulated in survivors (including multipath genes *tnfa, akt3a, nfkb2, chuk, mapk14a, il1b, rac1, ifnphi3, traf6, ifng1-2, map3k7*), suggesting that first might be related to viral shutoff while the former might be related to host survival. However, did survivors resulted from infection and clearing the virus, or were the fish never infected from the beginning? . It is unlikely that any fish remained unexposed to the virus given their high dosage (5-9 x 10^7^ pfu/ml) and the long exposure time (90 min) used for the infections. Furthermore, in the described SVCV infections as well as in many other in our laboratory under similar conditions, symptoms of infection did appeared in a few fish as early as after 3-days and in 80-90% of the fish after 5-7-days. Mortalities also began after 3-days, leaving 30-40% of survivors after 30-days. We concluded that it is reasonable to work under the assumption that fish sampled for RNA in microarray analysis were all infected at the beginning or at least exposed to the virus. However, even though all fish were exposed, they might still resist infection from the beginning, for instance because of their previous high levels of innate defenses or because other genetic possibilities. On the other hand, the earliest responses could have been initiated and resolved before 2-days of infection and therefore would remain undetected by the studies reported here. Evidences to favor any of those alternative explanations would require the follow up individual fish and remain difficult to be solved without killing individual fish.

Independently of the mechanisms that could underlay their regulation, the differentially expressed multipath genes of Table 1 should be among the most important targets of SVCV infection. Therefore, multipath genes could be chosen as candidates to a possible search for common TFs and associated drugs. Because their regulation across many pathways should increase their impact simultaneously in many responses, multipath genes are likely to interlink the crosstalk in the complex pathway networks.

### Does SVCV infection causes transcriptional shutoff ?

SVCV infection caused inhibition (shutoff) in most pathways, probably to favor initial viral replication. That would confirm previous proposals that early viral infections were immunosuppressive to counteract fish innate responses [9] or to interfere with host protective apoptosis [47,48]. A similar downregulation in internal organs but not in fins, was reported in cold-acclimatized zebrafish infected-by-immersion with VHSV [16] in contrast to infection-by-injection in other fish/viral models [19,20,22-24,27-30,49-52]. The failure to generate protective immune responses to SVCV in zebrafish larvae reported before might be also due to viral shutoff [12]. Different mechanisms for shutoff of host responses have been described for many viruses [53-56], including inhibition of transcription by rhabdoviruses [57]. Fish rhabdoviral proteins most likely to induce shutoff could be the matrix (M of SVCV) and/or the non-viral (NV of *novirhabdoviruses*) proteins [9], but that remains to be further investigated. On the other hand, upregulation of inflammatory genes, such as *il1b* and *tnfa*, have been reported in zebrafish intraperitoneally injected with SVCV [36], in contrast to the downregulation found in this work. Most probably, these differences are also related to the infection route (injection versus immersion), to the different fish size (normal versus XL) or the different organs (whole head versus head kidney and spleen) used in both studies [36].

As mentioned above, for most of the pathways studied (except for the complement and coagulation cascade), more genes were downregulated after 2-days than in survivors while more genes were upregulated in survivors than after 2-days. However, most importantly the genes implicated in both cases were generally different. Years of competitive evolution between SVCV and fish should have provided virus with effective genes for shutoff of initial fish responses and individual fish with strong gene responses to partially survive viral exposure. By using bioinformatic tools to compare down and upregulated multipath gene promoters, we identified putative transcription factors (TFs) which were different for downregulated (*pax6, dmrt2a*, and *pou2*) and upregulated (*nkx1.2l, pou1f1, hoxa, hoxd, bcl11a, foxb1.2, foxd3, foxl, foxq*) multipath genes (Table 2). In addition, experimental results showed that some of those common TFs were differentially regulated after 2- or 30-days, suggesting that down and upregulated multipath genes might be independently controlled. However more experimental evidence should be obtained on these preliminary results to confirm such hypothesis and/or to identify other TFs. Most likely the identified TF are only part of the gene regulators of multipath genes, because of the strict criteria used to extract TFBS from the abundant list of possibilities and because more TF could have been included in the targeted microarray if other sources of TF sequences had been included in the original design (ie. http://www.geneontology.org/GO.downloads.annotations.shtml). Work searching for additional TF probes is ongoing.

### Transcript and protein expression levels

While the induction of *nfkb2* by viral infection is something that has been reported for many viruses and in many host species, its involvement in the host-response to SVCV had not been previously reported. To validate some of those transcription results, we focused on the *nfkb* family of proteins since *nfkb2* was one of the more pleiotropic multipath genes identified. Because an increment in *nfkb2* transcription does not necessarily mean that the *nfkb2* protein was translocated to the nuclei of the cells to activate other genes, we studied possible *nfkb2* protein translocation by means of *in vitro* immunofluorescence assays. Results not only showed that the *nfkb2* protein translocated to the nuclei but also increased after SVCV infection. Furthermore, the increase of immunofluorescence detected after SVCV infection confirms microarray data. Together with the unchanged transcription of *nfkb2* and downregulation of *nfkbiab* after 2-days *in vivo*, transcript/protein changes could be explained by assuming that increased *nfkb2* protein levels were due to translation of preexisting mRNA and that downregulation of *nfkbiab* will favor nuclear translocation of the *nfkb2* protein.

A full understanding of infection-related changes will require a reliable quantitative assessment of all possible protein changes. In the future, it is expected that new proteomic methods based on liquid phase chromatography and targeted mass spectrophotometry could be applied to fish. Such targeted techniques are actually being rapidly developed to allow for the simultaneous analysis of many proteins together with their possible post-translational modifications with higher sensibility and reproducibility than before [58,59]. 

### Identification of candidate drugs affecting differentially expressed multipath genes

Our initial hypothesis being that there might be drugs to prevent seasonal SVCV-infection, we further hypothesized that putative drug targets could be among the multipath genes. Thus, drug candidates targeting multipath genes could be identified on human drug databases (DrugBank database, version 3.0 (http://www.drugbank.ca, accessed February 2013) [60]. For instance, drugs could be used to activate multipath genes which were downregulated after 2-days such as *akt3* (minoxidil, temsirolimus) or *mapk14a* (bromocriptine, carbegoline), to study the effects of activating (thymalfasin) or inhibiting (minocycline) *nfkb2* on the outcome of SVCV infections, to activate *ifnphi3* (imiquimod) or *ifng1-2* (thymalfasin), etc. Associated drugs that could interfere with the identified common TFs (Table 2) would be most efficacious to control responses to SVCV infection, however we could not find any drugs associated with them. The zebrafish model with its possibilities of easy handling, abundance of genetic strains and genetic-related methods opens a wide variety of possibilities for high throughput screening of novel drug derivatives.

## Supporting Information

File S1 **Figures S1 – S5.** Figure S1.“Natural killer cell-mediated cytotoxicity” of zebrafish genes after SVCV infection. The gene boxes (zebrafish gene short names in *italics*) and their relationships obtained from the corresponding human KEGG-pathway correspond to tested gene transcripts of the pathway section of the targeted microarrays. Capital letters, inputs (black) and outputs (blue). Vertical parallel lines, cellular membranes of healthy or infected cells (left) and natural killer cells (right). Continuous black and blue arrows, activation between gene products. Discontinuous black arrows, inhibition between gene products. Gray form, healthy cell pathway. Folds were calculated as described in methods by the formula, fluorescence value 2- or 30-days after infection / fluorescence mean in non-infected controls. Gene short names surrounded by an square, multipath genes. Red *italic* letters, downregulated transcripts with folds <0.5. Orange *italic* letters, downregulated transcripts with folds <0.66. Bright green *italic* letters, upregulated transcripts with folds >2. Dark green *italic* letters, upregulated transcripts with folds >1.5. Black *italic* letters, not differentially expressed. A, 2-days after SVCV infection. B, 30-days after SVCV infection (survivors). Figure S2, “Apoptosis” pathway of zebrafish genes after SVCV infection. Underlined, Extrinsic and intrinsic path inputs. Gray form, survival pathway. Red *italic* letters, downregulated transcripts with folds <0.5. Orange *italic* letters, downregulated transcripts with folds <0.66. Bright green *italic* letters, upregulated transcripts with folds >2. Dark green *italic* letters, upregulated transcripts with folds >1.5. Black *italic* letters, not differentially expressed. A, 2-days after SVCV infection. B, 30-days after SVCV infection (survivors). Other details as described in the legend of Figure S1 in File S1. Figure S3, “B-cell receptor signaling pathway” of zebrafish genes after SVCV infection. Gray form, pathway triggered by the presence of antigen. Red *italic* letters, downregulated transcripts with folds <0.5. Orange *italic* letters, downregulated transcripts with folds <0.66. Bright green *italic* letters, upregulated transcripts with folds >2. Dark green *italic* letters, upregulated transcripts with folds >1.5. Black *italic* letters, not differentially expressed. A, 2-days after SVCV infection. B, 30-days after SVCV infection (survivors). Other details as described in the legend of Figure S1 in File S1. Figure S4, “T-cell receptor signaling pathway” of zebrafish genes assayed after SVCV infection. Gray form, main pathway. Red *italic* letters, downregulated transcripts with folds <0.5. Orange *italic* letters, downregulated transcripts with folds <0.66. Bright green *italic* letters, upregulated transcripts with folds >2. Dark green *italic* letters, upregulated transcripts with folds >1.5. Black *italic* letters, not differentially expressed. A, 2-days after SVCV infection. B, 30-days after SVCV infection (survivors). Other details as described in the legend of Figure S1 in File S1. Figure S5, "MAPK signaling pathway" of zebrafish genes after SVCV infection. Gray form, classical MAPK path triggered by different kinds of stress (rest of paths belong to the JNK, p38 and ERK5 alternative MAPK paths). MAP4K phosphorylates > MAP3K which phosphorylates > MAP2K which phosphorylates > MAPK which phosphorylates >TF (transcription factor). Red *italic* letters, downregulated transcripts with folds <0.5. Orange *italic* letters, downregulated transcripts with folds <0.66. Bright green *italic* letters, upregulated transcripts with folds >2. Dark green *italic* letters, upregulated transcripts with folds >1.5. Black *italic* letters, not differentially expressed. A, 2-days after SVCV infection. B, 30-days after SVCV infection (survivors). Other details as described in the legend of Figure S1 in File S1.
(ZIP)

Table S1 **List of primers designed for the qPCR of multipath genes.** Microarray analysis results of the differentially expressed multipath genes were validated by RTqPCR (see methods) by using their reference accession numbers from the microarray design to search suitable primers with the Array Designer 4.3 program (Premier Biosoft, Palo Alto CA, USA). Forward and reverse primers amplifying 100-120 bp were designed. The list contains 16 differentially expressed multipath genes corresponding to Table 1, *mapk10* as a non-differentially expressed multipath gene control and *rplp0* as a normalizer gene.
(DOCX)
