# Supplementary material for: Identification of Multipath Genes Differentially Expressed in Pathway-Targeted Microarrays in Zebrafish Infected and Surviving Spring Viremia Carp Virus (SVCV) Suggest Preventive Drug Candidates
Source: PLoS One. 2013 Sep 12;8(9):e73553. doi: 10.1371/journal.pone.0073553 (PMC3772095; doi:10.1371/journal.pone.0073553)
Supplement: Table S1 — List of primers designed for the qPCR of multipath genes. Microarray analysis results of the differentially expressed multipath genes were validated by RTqPCR (see methods) by using their reference accession numbers from the microarray design to search suitable primers with the Array Designer 4.3 program (Premier Biosoft, Palo Alto CA, USA). Forward and reverse primers amplifying 100-120 bp were designed. The list contains 16 differentially expressed multipath genes corresponding to Table 1, mapk10 as a non-differentially expressed multipath gene control and rplp0 as a normalizer gene. (DOCX) [file pone.0073553.s002.docx]

**Table S1**

**List of primers designed for the qPCR of multipath genes**

| **Gene** | **Accession** |  |  |
| --- | --- | --- | --- |
| **name** | **number** | **5' > 3’ Forward** | **5' > 3’ Reverse** |
|  |  |  |  |
| *rplp0* | NM_131580 | CACGCTGCTGAACATGCTGAAC | AATCCTCCTTGGGTGCCTCCTC |
|  |  |  |  |
| *tnfa* | NM_212859 | TGCTGCCGTCTGCTTCAC | AGTGCTGTGGTCGTGTCTG |
| *akt3a* | NM_001197201 | CCGCCTATGCTTCGTGATG | CTTCTCGTGGTCCTGATTGTAG |
| *pik3r5* | XM_002662345 | TGTGTCGTAACCTGCTCTCC | GCCAACTCATCATCGCTAAGG |
| *nfkb2* | NM_001001840 | AGGTCAGTATCCAGTCCATCTC | TCCAGCAGCAGGTCTCTTC |
| *chuck* | NM_200317 | GACTACAGCCGCCTCTTCC | GACGACGAGCATAAGGACTTC |
| *nfkbiab* | NM_199629 | CCGTCGCTTGAGGAATGC | CTGGAGGATGGCTGAGAGG |
| *map2k1* | NM_213419 | TCAAGGCACTCACTACTCTGTC | CACAACCATCCAGCGAAGTC |
| *mapk14a* | NM_131722 | CACTCGCATTCCAAGCAGAC | CCAGCAAGCCGATCACATTC |
| *il1b* | NM_212844 | ATCCAAGAGCGTGAAGTGAAC | CATCTCCACCATCTGCGAATC |
| *rac1* | NM_199771 | ATTCCTGCTGCCTTCTAATGAC | ACTGCCAGACTCCTCCAATAG |
| *raf1b* | NM_001199755 | ATTATCCGACGATGGCAGAATC | CAGCAGGAGTTGGCGAATG |
| *ifnphi3* | NM_001111083 | GGACCTATACACTTCTGGAGAC | CAATGCGTCAATGTTCTGGAG |
| *ifn1-2* | NM_212864 | AGAATGACAGCGTGGATGAAG | TAGCCTGCCGTCTCTTGC |
| *hras* | XM_001923404 | ATCGCCTGGAGAGTGACTG | TGGTTCTGGATAAGTTGGATGG |
| *map3k7* | NM_001020750 | AGAGGCAGCAGCGTAGAG | GTCTGTCCTGTCTGGTGTAATC |
| *traf6* | NM_001044752 | GCTCCTCGCTGTTCTAACTTC | CAGGTCTGGCTTGGTCTCC |
|  |  |  |  |
| *mapk10* | NM_001037701 | ATCCAGCGAAGCGAATATCAG | AGGCAGGCGGTTAGTCAC |
|  |  |  |  |
